# Associations between parent-child relationship, self-esteem, and resilience with life satisfaction and mental wellbeing of adolescents

**DOI:** 10.3389/fpubh.2023.1012337

**Published:** 2023-01-24

**Authors:** Vu Anh Trong Dam, Ha Ngoc Do, Thao Bich Thi Vu, Khanh Long Vu, Hoang Minh Do, Nga Thu Thi Nguyen, Tham Thi Nguyen, Thuc Minh Thi Vu, Thao Phuong Thi Nguyen, Pascal Auquier, Laurent Boyer, Guillaume Fond, Carl A. Latkin, Cyrus S. H. Ho, Roger C. M. Ho

**Affiliations:** ^1^Institute for Global Health Innovations, Duy Tan University, Da Nang, Vietnam; ^2^Faculty of Nursing, Duy Tan University, Da Nang, Vietnam; ^3^Youth Research Institute, Ho Chi Minh Communist Youth Union, Hanoi, Vietnam; ^4^Vietnam Youth Academy, Hanoi, Vietnam; ^5^Faculty of Social Sciences and Humanities, Hanoi Metropolitan University, Hanoi, Vietnam; ^6^Institute of Health Economics and Technology, Hanoi, Vietnam; ^7^EA 3279, CEReSS, Research Centre on Health Services and Quality of Life, Aix Marseille University, Marseille, France; ^8^Bloomberg School of Public Health, Johns Hopkins University, Baltimore, MD, United States; ^9^Department of Psychological Medicine, Yong Loo Lin School of Medicine, National University of Singapore, Singapore, Singapore; ^10^Institute for Health Innovation and Technology (iHealthtech), National University of Singapore, Singapore, Singapore

**Keywords:** multilevel predictors, mental wellbeing, satisfaction with life, youth, adolescent, Vietnam

## Abstract

**Purpose:**

Mental wellbeing and life satisfaction play an important role in the development of adolescents, yet factors potentially influencing these states have not been sufficiently studied, especially in Vietnam. This study aims to fill the research gaps by exploring the associations of the parent-child relationship, self-esteem, and resilience on the mental wellbeing and satisfaction with life of adolescents.

**Methods:**

A cross-sectional study was conducted from June to July 2020 on 1,023 adolescents from 10 to 18 years old living in Vietnam. To assess the satisfaction with life as well as the mental wellbeing of participants, this study used the Satisfaction with life and The World Health Organization-Five Wellbeing Index scale.

**Results:**

More than 70% of participants reported having conflicts with their parents (74.6% of those conflicted with their father ad 73.9% of those conflicted with their mother), while 26.3% stated dissatisfaction with life. The mean score of mental wellbeing was 61.5 (SD = 23.0). Higher academic performance, self-esteem, resilience, encounter loneliness and isolation within own family, and having support and sharing from family members had a positive effect on life satisfaction and mental wellbeing. Female participants had higher satisfaction with life score (Coef = 0.77; 95%CI = 0.10; 1.44) but they had a lower mental wellbeing score (Coef = −6.00; 95%CI = −8.57; −3.44) than male participants. High school students had lower both satisfaction with life and mental wellbeing scores than secondary students.

**Conclusion:**

The results highlight the importance of being aware of the influence that expectations (of higher grades) and bias (toward male children) imposed by parents, teachers, and society on the mental wellbeing of youths, especially in Asian cultures. Strengthening the family bond and encouraging young people to share their feeling is also crucial to enhancing the mental health condition of adolescents.

## Introduction

Mental wellbeing, as an integral part of our overall health, is considered a vital public health issue. It encompasses multiple dimensions, such as our performance in relationships, work, and entertainment activities in the face of adversity (1). Mental wellbeing is defined as positive mental health, in which every individual realizes their potential, can cope with daily stressors, works effectively, and contributes to their community (2). Among the components of subjective wellbeing, life satisfaction has been considered one of the most important indicators (3). According to Diener et al. life satisfaction has been defined as “a person's cognitive and affective evaluations of his or her life,” it was also the main dimension of the mental health (4, 5). Assessing the life satisfaction and mental wellbeing of adolescents is considerably more challenging than with other age groups, as the transition from childhood to maturity presents its unique set of problems and opportunities (6). Several studies revealed a strong correlation between life satisfaction and quality of life, family environment, self-esteem physical, and mental health (4, 7–9). In addition, higher satisfaction with life was likely to play an important role against negative mental health situations such as stress, and prevent the development of violent behavior or suicidal and self-harm behavior (10–13). Suldo and Huebner also discovered that teenagers with high levels of life satisfaction were less likely to engage in future-threatening activities stresses (13).

The most common factors associated with adolescents' life satisfaction include self-esteem, internal control, family, and peer relationships (14). Although individuals may form many interpersonal relationships throughout their lives, the parent-child relationship is especially critical to the development and success of adolescents as its influences begin early in life (15). Therefore, it is important to determine how different family dynamics influence an adolescent's life satisfaction. Current research suggested that teenage life satisfaction is linked to a variety of family factors, such as parental support and family connections (16, 17). Adolescents tend to have lower life satisfaction when there are conflicts and high stress within the family (16). On the other hand, research by Moskenes revealed that self-esteem is a positive factor associated with adolescents' life satisfaction and mental wellbeing (14). Self-esteem is defined as a person's perception of sentiments and their value in the community (18) and has been linked with several positive outcomes, including psychological health and wellbeing of adolescents (17, 19, 20). The definition of resilience covers the dynamic process of positive adaptation to adversity and stress, and is usually included in research on children or public health patients (21, 22). Among adolescents, resilience has a major impact on academic achievement, social competence, and the avoidance of hazardous conducts, thus it is strongly associated with adolescent life satisfaction (23, 24). This study identifies parent-child relationship, self-esteem, and resilience as the three most important indicators of life satisfaction and mental wellbeing and will assess the correlation of these concepts with proposed problems as well as with each other.

In Vietnam, existing literature suggested that adolescents are increasingly exposed to risk factors such as school violence, mental disorders, juvenile delinquency, and abortion (25). A recent report by UNICEF indicated that mental health disorders among young people in Vietnam are rapidly increasing, with 8–29% experiencing mental health problems (26). Gender discrimination also exacerbates this problem: in Vietnamese tradition, boys tend to receive certain privileges and more parental attention than girls do (27, 28). This dynamic deeply upsets the relationship between children and parents, and lowers the female child's self-esteem, especially in their adolescent years. Despite the severity of problems among adolescents, studies on life satisfaction and mental wellbeing of this population in Vietnam have been limited, especially those that investigate the influence of the parent-child relationship on the overall life satisfaction and mental wellbeing of children in their adolescent years. Therefore, this study aims at filling the research gap and determining patterns of life satisfaction and mental health of adolescents in Vietnam as well as exploring the potential impact of parent-child relationships, self-esteem, and resilience of Vietnamese young people on their life satisfaction and mental wellbeing. The main hypothesis of this study is that the parent-child relationship has a significant role in Quality of Life and mental wellbeing through self-esteem and protective factors for resilience as a mediator.

## Methods

### Study setting and participants

We conducted online cross-sectional research in four Vietnamese provinces (Hung Yen, Can Tho, Thanh Hoa, and Ba Ria–Vung Tau) in Vietnam, from June to July 2020. These were Vietnam's major cities and provinces, all of which were undergoing rapid urbanization. Participation criteria were: (1) currently studying grades 6 to 12; (2) currently residing in Vietnam, and (3) willing to participate in this study by online informed consent. Participants who had major diseases or were unable to answer questions were excluded from the recruiting procedure. We used the convenience sampling approach in four steps as (1) the research team started with core groups of student union presidents at schools; (2) these core groups received a link to a website containing introductory information about the study and the questionnaire; (3) they prompted to forward it to others in their network by email, social media, or other online means; and (4) invitees continued to complete surveys and encourage others to do so as well. At the end of the data collection period, there were 1,023 students from grades 6 to 12 completed the survey.

### Measurement and instrument

We developed a structured questionnaire and uploaded it onto the online survey platform SurveyMonkey (surveymonkey.com). This approach was adopted as it was economical, time-saving, user-friendly for teenagers, and effective in reaching a large sample. The questionnaire includes four major components: (1) general characteristics (of participants); (2) Satisfaction with Life among participants; (3) Relationship in the family of participants (4) Social support, the Protective Factors for Resilience scale (PFRS), and Self-esteem Scale. The survey was originally piloted with a small sample of students to confirm that the translated instruments in Vietnamese were cross-culturally valid. The updated questionnaire was then posted to the online survey platform. Data collection commenced after the online survey system was thoroughly tested to assure the accuracy of the survey contents and that no technical issues occurred.

### Socioeconomic characteristic

We asked participants to report information regarding their gender, education, living location, people living together, academic performance, and the age of their father/mother. In terms of academic performance, in Vietnam, the academic performance of students was divided into four levels based on 10-scale: moderate and below (<7), fair (from 7 to <8), good (from 8 to <9), and excellent (equal or more than 9). Regarding level of education, we had three levels of education: Primary school (grade 1–5 correspond to 6–10 years old), secondary school (grade 6–9 correspond to 11–14 years old), and high school (grade 10–12 correspond to 15–17 years old). In this study, we defined a nuclear family as a family that had two generations such as parent and their child.

### Satisfaction with life scale

The SWLS measures a person's overall happiness in life. This measure has five items, and participants respond to each question on a seven-point Likert scale ranging from one (strongly disagree) to seven (strongly agree) (29). The possible range of scores is 5–35, divided into 7 levels of satisfaction with life from 5 to 9 points “Extremely dissatisfied” to include 31–35 points “Extremely satisfied” (30). The Cronbach's alpha was 0.83.

### The World Health Organization-Five Wellbeing Index

The WHO-5 is a brief self-reported assessment of present mental wellbeing developed by the WHO. This scale, which is appropriate for children aged 9 and up, has been proven to have sufficient validity in screening for depression and assessing outcomes in treatment studies (31). It consists of five statements for which respondents were given a 6-Likert scale ranging from 0 (All of the time) to 5 (At no time) (31). The WHO-5 raw score varies from 0 to 25, and the final score is calculated by multiplying the raw score by 4, with 0 indicating the lowest possible wellbeing and 100 being the highest possible wellbeing (31). The Cronbach's alpha was good at 0.86.

#### The relationship between parents and children

Three questions were used to access the relationship between parents and children such as “Do you have conflicts with your father,” and “Do you have conflicts with your mother” with 2 answer options (Yes/No), and “How many family members can you confide in.”

#### The feeling of the students at home

We employed 8 questions to explore the connection with family members and the feeling of loneliness at home. These questions included:

*Encounter loneliness and isolation within own family:*

1) Do not feel the family member is a good companion.
2) Feeling alone even at home.
3) My family member is by my side but does not care about me.
4) Feeling no connection to my family.
5) Feeling family relationships have less meaning to me.

*Having support and sharing from a family member*.

6) Feeling part of the family.
7) Family members are always willing to listen to and understand me.
8) Family members are always ready to encourage and comfort me.

Each item was marked from 1 (Never) to 5 (Always). The total score of “*Encounter loneliness and isolation within own family”* and “*Having support and sharing from a family member”* was reversed from 1 to 10. Participants who had higher “*Encounter loneliness and isolation within own family”* scores indicated a stronger feeling of loneliness and isolation, by contrast, those who had higher “*Having support and sharing from a family member”* scores indicated better the connection with family members and the feeling of loneliness at home.

### Protective factors for resilience scale

*PFRS* includes 15 categories in 3 domains: Personal Resource, Social Resources–Peers, and Social Resources–Family (5 items per domain) (32). A 7-Likert scale was used to respond to each question (from 1-strongly disagree to 7-strongly agree) (32). The total score of each domain was calculated by the sum score of each item. Participants who had a higher score reflected the sense of having more personal and psychosocial (from family and peers) protective factors available to be resourceful in the face of adversity (32). The Cronbach's alpha was good at 0.92.

### Self-esteem scale

*Rosenberg's self-esteem scale (RSE scale)*: This is a 10-item measure that assesses one's overall self-worth by assessing both positive and harmful feelings toward oneself (33). The 10 RSE elements are usually rated on a 4-point Guttman-type scale by researchers (33). Researchers frequently include a neutral response choice, resulting in a five-point Likert-type response scale ranging from Strongly Disagree (0) to Strongly Agree (4). In this study, a 5-Likert was used, and the total score of the 10 items was used to assess the self-esteem of objectives, with scores ranging from 0 to 40. Items 2, 5, 6, 8, and 9 have a score of 0 indicating Strongly Disagree, 1 = Disagree, 2 = Neutral 3 = Agree, and 4 = Strongly Agree). Higher scores indicate higher self-esteem (33). The Cronbach's alpha was 0.64.

### Data analysis

We analyzed data using STATA version 16 (Stata Corp. LP, College Station, United States of America). To clean data before analysis, we used the Listwise Deletion method, there were 1,222 participants recruited in this study, and 1,023 students from grades 6–12 completed the survey. Hence, the data of 199 people were deleted, and the response rate was 83.72%. The mean and standard deviation (SD) were used to describe continuous data, whereas frequencies and percentages were used to represent categorical variables. To see if there are any variations in participants' life satisfaction and mental wellbeing based on specific variables, Wilcoxon rank-sum test, Kruskal-Wallis test for continuous variables, and χ2 test for categorical variables were used.

Potential covariates for full models of life satisfaction and mental wellbeing among Vietnamese students included individual characteristics, relationships in the family, and protective factors for resilience. Multivariate Tobit regression was conducted to determine factors related to life satisfaction as well as mental wellbeing among Vietnamese students. These models were then combined with the stepwise forward strategies to produce reduced models with *p* < 0.2 as the threshold for included variables. The *p*-value (P)<0.05 was considered statistically significant.

The following were used in the goodness-of-fit tests for the hypothesis model: goodness-of-fit index (GFI), comparative fit index (CFI), root mean squared error of approximation (RMSEA), Tucker–Lewis index (TLI), and the standardized root mean squared residual (SRMR). The results showed that the structure had a good fit with RMSEA (0.049), CFI (0.994), and SRMR (0.014).

### Ethics approval and consent to participate

All procedures performed in studies involving human participants were by the ethical standards of the Youth Research Institute, Vietnam, and with the 1964 Helsinki Declaration and its later amendments or comparable ethical standards. Online Informed consent was obtained from all participants. Informed consent was also obtained from the participants' parents.

## Results

Table 1 shows the individual characteristics of 1,023 students in this study. About 50.4% of the participants were female. Most of the respondents were living in rural areas (73.7%) and had a nuclear family (81.1%). Good or fair academic performances were the most popular responses among participants (44.6 and 24.8%, respectively). Additionally, the percentages of having a conflict with their father, their mother, and their parent were 74.6, 73.9, and 62.5, respectively. The mean ages of students' mothers and fathers were 42.0 and 45.2, respectively. The mean score of the three subscales of the resilience scale “Personal resources,” “Social resources-peer,” and “Social resources-family” was 25.2, 24.7, and 28.7, respectively. Besides, the average self-esteem score was 22.4. In terms of life satisfaction, There were 67.7% of participants reported that they felt from “Slightly satisfied” to “Extremely satisfied” with life. The other levels of life satisfaction extremely dissatisfied, dissatisfied, slightly dissatisfied, and neutral, had a smaller percentage with 1.9, 7.3, 17.1, and 6.1%, respectively, and there was a statistically significant difference between male and female respondents (*p* < 0.05).

**Table 1:** Demographic characteristics and protective factors for resilience.

**Characteristics**	**Gender**				**Total**		***p*-value**

	**Male**		**Female**				
	***n***	**%**	***n***	**%**	***n***	**%**	
**Total**	507	49.6	516	5.4	1,023	100.0	
**Education**							
Secondary school	287	56.6	258	5.0	545	53.3	0.03
High school	220	43.4	258	50.0	478	46.7	
**Location**							
Rural	389	76.7	365	70.7	754	73.7	0.03
Urban	118	23.3	151	29.3	269	26.3	
**Current living**							
Nuclear family	398	78.5	432	83.7	830	81.1	0.06
Extended family	89	17.6	73	14.2	162	15.8	
Others	20	3.9	11	2.1	31	3.0	
**Academic performance**							
Excellent (≥9/10)	105	20.7	99	19.2	204	19.9	0.02
Good (8– < 9/10)	204	40.2	252	48.8	456	44.6	
Fair (7– < 8/10)	132	26.0	122	23.6	254	24.8	
Moderate and below (< 7/10)	66	13.0	43	8.3	109	10.7	
**Conflict with father**							
Yes	394	77.7	369	71.5	763	74.6	0.02
No	113	22.3	147	28.5	260	25.4	
**Conflict with mother**							
Yes	382	75.4	374	72.5	756	73.9	0.30
No	125	24.6	142	27.5	267	26.1	
**Conflict with both parents**							
Yes	327	64.5	312	60.5	639	62.5	0.18
No	180	35.5	204	39,5	384	37.5	
**The number of family members can confide in**							
None	22	4.3	6	1.2	28	2.7	0.01
One	343	67.7	373	72.3	716	70.0	
Two or more	142	28.0	137	26.6	279	27.3	
**Satisfaction with life**							
Extremely dissatisfied	17	3.4	2	0.4	19	1.9	< 0.01
Dissatisfied	31	6.1	44	8.5	75	7.3	
Slightly dissatisfied	92	18.2	83	16.1	175	17.1	
Neutral	30	5.9	32	6.2	62	6.1	
Slightly satisfied	132	26.0	103	20.0	235	23.0	
Satisfied	112	22.1	152	29.5	264	25.8	
Extremely satisfied	93	18.3	100	19.4	193	18.9	
	**Mean**	**SD**	**Mean**	**SD**	**Mean**	**SD**	***p*** **-value**
**Father's age**	45.7	6.1	44.6	6.1	45.2	6.1	< 0.01
**Mother's age**	42.3	5.7	41.6	5.3	42.0	5.5	0.05
**Protective factors for resilience scale (range: 5–35)**							
Personal resources	25.5	5.2	25.0	5.3	25.2	5.3	0.08
Social resources–peers	24.9	5.5	24.6	5.7	24.7	5.6	0.82
Social resources–family	28.7	5.1	28.6	6.2	28.7	5.7	0.22
**Self-esteem scale (range: 0–40)**	22.2	5.2	22.6	4.9	22.4	5.0	0.08

The construct validity and reliability of the relationship in family participants were reported in Table 2. Based on factor analysis, 8 items were divided into 2 factors, including “Encounter loneliness and isolation within own family” (5 items), and “Having support and sharing from family members” (3 items) with Cronbach's alpha for each factor was 0.77. Besides, the proportion of students who selected “very often” for each item of factor 1 was from 42.1 to 58.6%. Similarly, the figure for the three remaining questions ranged from 25.9 to 55.5%.

**Table 2:** Factor loadings of student's feeling at home of participants.

**Variable**	**Very often (n)**	**Very often (%)**	**Encounter loneliness and isolation within own family**	**Having support and sharing from a family member**
1. Do not feel the family member is a good companion	541	52.9	0.5045	
2. Feeling alone even at home	362	35.4	0.5573	
3. My family member is by my side but does not care about me	265	25.9	0.7047	
4. Feeling no connection to my family	568	55.5	0.7406	
5. Feeling family relationships have less meaning to me	431	42.1	0.4077	
6. Feeling part of the family	599	58.6		0.3713
7. Family members are always willing to listen to and understand me	548	53.6		0.8178
8. Family members are always ready to encourage and comfort me	554	54.2		0.7960
**Cronbach's alpha**			0.75	0.75
**Mean**			3.7	7.5
**SD**			1.5	2.0

Table 3 reveals the mean score of satisfaction with life scale and mental wellbeing of participants regarding some characteristics. Secondary students had higher life satisfaction as well as mental wellbeing score than high school students. The mean scores of students with excellent academic performance in both life satisfaction and mental health were higher than others (*p* < 0.05). There was a statistically significant dissimilarity in the score for life satisfaction and mental wellbeing between people having conflict with their father, their mother, or their parent as well as the number of their family members that they can confide in. Participants who had no conflict with their father/mother/both parents or had people to confide in had higher scores for satisfaction with life and mental wellbeing than others. The value score of the life satisfaction scale and WHO-5 scale was about 23.9 points and 61.5 points, respectively.

**Table 3:** The SWLS and WHO-5 of participants regarding different characteristics.

**Characteristics**	**Satisfaction with life (range: 5–35)**			**Mental wellbeing (range: 0–100)**		

	**Mean**	**SD**	***p*** **-value**	**Mean**	**SD**	***p*** **-value**
**Total**	23.9	6.8		61.5	23.0	
**Gender**						
Male	23.5	7.0	0.08	63.9	22.0	< 0.01
Female	24.3	6.6		59.3	23.6	
**Education**						
Secondary school	24.7	7.1	< 0.01	65.0	24.3	< 0.01
High school	23.0	6.3		57.6	20.6	
**Location**						
Rural	24.0	6.9	0.25	61.3	23.0	0.56
Urban	23.5	6.5		62.3	22.7	
**Current living**						
Nuclear family	23.7	6.8	0.06	61.4	23.0	0.07
Extended family	24.4	7.2		60.7	21.8	
Others	26.2	5.8		70.6	25.0	
**Academic performance**						
Excellent (≥9/10)	25.2	6.5	0.01	64.4	25.7	< 0.01
Good (8– < 9/10)	23.8	6.5		61.6	21.0	
Fair (7– < 8/10)	23.5	7.0		60.9	24.3	
Moderate and below (< 7/10)	22.6	7.8		57.4	21.7	
**Conflict with father**						
No	25.3	7.0	< 0.01	66.2	24.8	< 0.01
Yes	23.4	6.7		59.9	22.1	
**Conflict with mother**						
No	25.9	6.2	< 0.01	67.0	22.8	< 0.01
Yes	23.2	6.9		59.6	22.7	
**Conflict with both parents**						
No	25.2	6.5	< 0.01	65.9	23.0	< 0.01
Yes	23.1	6.8		58.9	22.5	
**The number of family members can confide in**						
0	18.1	5.3	< 0.01	47.1	20.9	< 0.01
1	23.4	6.8		61.4	23.6	
≥2	25.8	6.5		63.3	21.1	

Table 4 showed results on factors associated with life satisfaction and mental wellbeing among participants. Females had a higher satisfaction with life score (Coef. = 0.77; 95%CI: 0.10; 1.44) but lower mental wellbeing score (Coef. = −6.00; 95%CI: −8.57; −3.44) than males. Those who were high school students and had conflicts with their fathers were likely to have lower life satisfaction points than others. Similarly, their figure for mental wellbeing was also lower. The score of satisfaction with life and mental wellbeing tended to increase by about 0.07 points and 0.56 points, respectively, when their father's age increased by 1 year. By contrast, students' mental wellbeing decreased by 0.77 when the age of the mother increased by 1 year. Besides, students' feelings at home were significantly associated with life satisfaction as well as the mental wellbeing of participants. “Encounter loneliness and isolation within own family” was a harmful factor of life satisfaction (Coef. = −1.05; 95%CI: −1.32; −0.79) and mental wellbeing (Coef. = −1.53; 95%CI: −2.51; −0.55). By contrast, “Having support and sharing from their family member” was a positive factor of life satisfaction (Coef. = 0.44; 95%CI: 0.23; 0.65). Those having family members to confide in intended to have a higher score of satisfaction with life than people who did not. When three domains of protective factors for the resilience scale–Personal Resources, Social Resources–Peers, and Social Resources–Family increased by one point, the satisfaction with life increased by 0.16 points, 0.22 points, and 0.16 points, respectively. Mental wellbeing scores also tended to increase by 0.83 points when the domain social resources-peer increased by one point. Meanwhile, self-esteem played a protective factor that increased both the life satisfaction and mental wellbeing of adolescents.

**Table 4:** Tobit regression for identifying factors related to satisfaction with life and mental wellbeing among students.

**Characteristics**	**Satisfaction with life**		**Mental wellbeing**	

	**Coef**.	**95%CI**	**Coef**.	**95%CI**
**Individual characteristics**				
**Gender (Male–Ref)**				
Female	0.77^**^	0.10; 1.44	−6.00^***^	−8.57; −3.44
**Location (Rural–Ref)**				
Urban	−0.59	−1.35; 0.18		
**Education (Secondary school–Ref)**				
High school	−1.79^***^	−2.53; −1.05	−7.95^***^	−10.74; −5.15
**Academic performance (Excellent–Ref)**				
Good (8– < 9/10)	0.60	−0.32; 1.53	5.76^***^	2.21; 9.30
Fair (7– < 8/10)	1.30^**^	0.23; 2.38	7.79^***^	3.71; 11.87
Moderate and below (< 7/10)	1.47^**^	0.16; 2.79	7.00^***^	1.98; 12.02
**Current living (Nuclear family**–**Ref)**				
Extended family			−2.91	−6.48; 0.66
Others			6.57^*^	−0.96; 14.11
**Age of father (Unit: year)**	0.07^**^	0.01; 0.13	0.56^***^	0.21; 0.91
**Age of mother (Unit: year)**			−0.77^***^	−1.15; −0.38
**Relationship in family**				
**Conflict with father (No**–**Ref)**				
Yes	−1.19^***^	−2.02; −0.36	−6.09^***^	−9.02; −3.16
**Conflict with mother (No**–**Ref)**				
Yes	−0.59	−1.44; 0.25		
**Student's feeling at home (unit: one score)**				
Encounter loneliness and isolation within own family	−1.05^***^	−1.32; −0.79	−1.53^***^	−2.51; −0.55
Having support and sharing from their family member	0.44^***^	0.23; 0.65		
**Number of family members can confide in (None**–**Ref)**				
One	1.29	−0.76; 3.33	7.73^*^	−0.06; 15.52
Two and above	2.20^**^	0.06; 4.35	7.26^*^	−0.87; 15.39
**Protective factors for resilience (unit: one score)**				
Social resources–family	0.16^***^	0.07; 0.26	0.27^*^	−0.05; 0.58
Social resources–peers	0.22^***^	0.14; 0.31	0.83^***^	0.53; 1.12
Personal resources	0.16^***^	0.06; 0.25		
**Self-esteem (unit: one score)**	0.16^***^	0.07; 0.24	1.58^***^	1.28; 1.87

*** p < 0.01,

** p < 0.05,

* p < 0.1.

**Figure 1:**
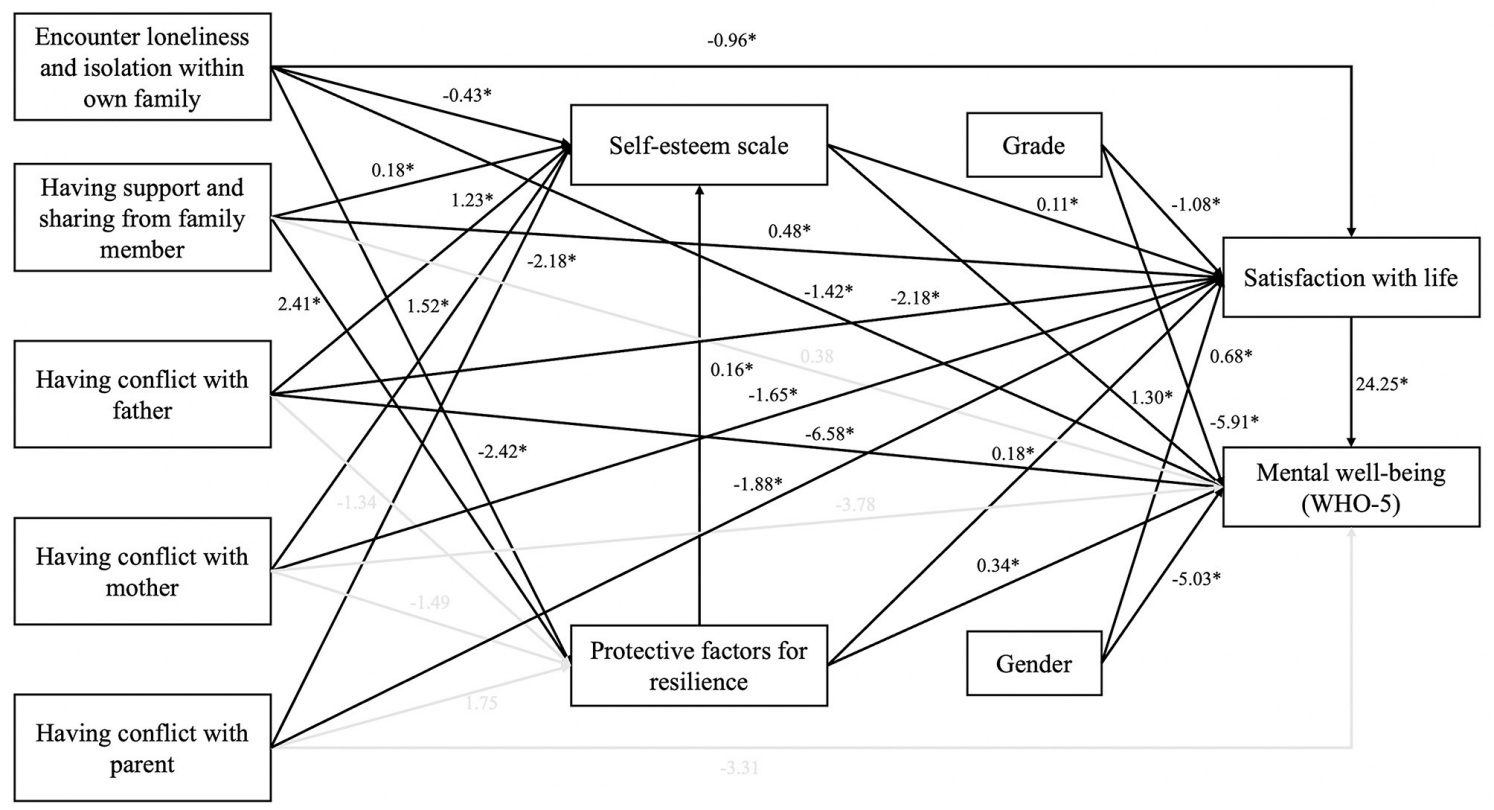
Structural equation modeling and standardized path coefficients (n = 1023) (*p < 0.05) (RMSEA = 0.051, CFI = 0.994, and SRMR = 0.013).

The path coefficients and *p*-value of structural equation modeling analysis are presented in Figure 1. The results showed that this structure had a good fit with RMSEA (0.051), CFI (0.994), and SRMR (0.013). Factors like “Having support and sharing from their family member,” “Self-esteem,” and “Protective factors for resilience” had positively associated with life satisfaction and mental wellbeing. By contrast, “Encounter loneliness and isolation within own family,” “Having a conflict with both parents” and “Grade” had negatively related to satisfaction with life, meanwhile, “Grade” and “Encounter loneliness and isolation within own family” had a negative correlation with mental wellbeing. Besides, “Having a conflict with father” were found to have a statistically significant relationship with both mental health and satisfaction with life. Overall, the model for mental wellbeing and satisfaction with life with ten factors explained 42.7% (R^2^ = 0.427) of the life satisfaction variance and mental wellbeing variance.

Table 5 presented the direct and total effects.

**Table 5:** Indirect effect for relationship between some characteristics and satisfaction with life as well as mental wellbeing mediated by self-esteem and protective factors for resilience.

**Pathways**	**Indirect effect**	**95% confident interval**	**Total effect/% total effect**	**Direct effect/% direct effect**
Conflict with father/self-esteem/Satisfaction with life	0.009	−0.001, 0.018	0.13/6.7%	0.139/6.2%
Conflict with father/self-esteem/WHO-5	0.030^*^	0.005, 0.056	0.094/32.0%	0.124/24.3%
Conflict with father/Resilience/Satisfaction with life	−0.015	−0.050, 0.019	0.155/10.0%	0.139/11.1%
Conflict with father/Resilience/WHO-5	−0.009	−0.028, 0.011	0.133/6.4%	0.124/6.9%
Conflict with mother/self-esteem/Satisfaction with life	0.011^*^	0.001, 0.021	0.095/11.4%	0.106/10.3%
Conflict with mother/self-esteem/WHO-5	0.038^*^	0.011, 0.065	0.034/110.3%	0.072/52.5%
Conflict with mother/Resilience/Satisfaction with life	−0.017	−0.053, 0.018	0.124/14.1%	0.106/16.4%
Conflict with mother/Resilience/WHO-5	−0.01	−0.030, 0.010	0.082/11.8%	0.072/13.4%
Conflict with both parents/self-esteem/Satisfaction with life	−0.017^*^	−0.032, −0.002	0.116/14.7%	0.134/12.8%
Conflict with both parents/self-esteem/WHO-5	−0.060^*^	−0.097, −0.022	0.010/583.8%	0.070/85.4%
Conflict with both parents/Resilience/Satisfaction with life	0.023	−0.027, 0.072	0.156/14.4%	0.134/16.8%
Conflict with both parents/Resilience/WHO-5	0.012	−0.015, 0.040	0.082/15.2%	0.070/17.9%
Having support and sharing /Self-esteem/Satisfaction with life	0.006	−0.001, 0.012	0.145/3.9%	0.140/4.1%
Having support and sharing /Self-esteem/WHO-5	0.020^*^	0.002, 0.038	0.054/37.5%	0.033/60.0%
Having support and sharing / Resilience /Satisfaction with life	0.129^*^	0.100, 0.157	0.268/47.9%	0.140/92.1%
Having support and sharing / Resilience /WHO-5	0.071^*^	0.046, 0.096	0.104/68.2%	0.033/214.4%
Encounter loneliness and isolation /Self-esteem/Satisfaction with life	−0.011^*^	0.019, −0.002	0.224/4.7%	0.214/4.9%
Encounter loneliness and isolation /Self-esteem/WHO-5	−0.037^*^	−0.055, −0.018	0.131/28.0%	0.094/38.9%
Encounter loneliness and isolation / Resilience /Satisfaction with life	−0.098^*^	−0.124, −0.072	0.312/31.5%	0.214/45.9%
Encounter loneliness and isolation / Resilience/WHO-5	−0.054^*^	−0.075, −0.034	0.148/36.6%	0.094/57.7%

Direct Effects: Significant direct effects were observed for conflict with both parents on satisfaction with life (β = 12.8%, *p* < 0.001) and mental wellbeing (β = 85.4%, *p* < 0.001) *via* self-esteem. Through self-esteem and resilience, both two factors “having support and sharing” and “encounter loneliness and isolation” were positively associated with life satisfaction and mental wellbeing.

Total effects: Conflict with both parents was negatively associated with life satisfaction *via* self-esteem (14.7% total effect on life satisfaction). Two constructs “Having support and sharing from family members,” and “Encounter loneliness and isolation within own family” influenced both satisfaction with life and mental wellbeing. In terms of the first construct, about 47.9 and 68.2% of the total effect on satisfaction with life and mental wellbeing respectively was indirect effect *via* the resilience scale, while these figures were 3.9 and 37.5% *via* self-esteem. Regarding the influence of the “Encounter loneliness and isolation” construct on life satisfaction and mental health, 4.7 and 28.0% of the total effect was indirect effect *via* the self-esteem scale, while 31.5 and 36.6% of the total effect was indirect effect *via* resilience scale.

## Discussion

This study revealed that a high percentage of children had a conflict with their father or mother (>70%), and nearly 30% of participants were dissatisfied with life. Conflict with father was the negative factor that decrease life satisfaction and mental wellbeing score. Encounter loneliness and isolation within own family and protective factor for resilience from personal, peers, and family had a positive impact on life satisfaction and mental wellbeing.

In terms of individual characteristics, our research reveals that male adolescents had higher life satisfaction scores than females. Gender differences in life satisfaction, though well-proven, are inconsistent. Research by Callahan et al. indicated that women tend to have a higher level of life satisfaction than men (33), while Graue et al. suggested that there is no gender difference in life satisfaction among their sample (34). Research on Vietnamese youths also reported a possibility of female children having lower study grades under the psychological influences of gender discrimination within their families (26, 35). Furthermore, when compared to adolescents with excellent academic performance, those with lower academic performance had higher life satisfaction and mental wellbeing scores. Indeed, the overemphasis on academic excellence has been found to have negative influences on the child's performance, as it leads to overwork, which often results in serious work-life imbalance (31). Thus, in Vietnam and other male-bias cultures, parents, teachers, and society must recognize and reduce the scope of such discrimination on life satisfaction and the mental wellbeing of adolescents.

This study revealed encounter of loneliness and isolation within own family had negatively associated with satisfaction with life and mental wellbeing. This result was similar to the Çivitci et al. (19). Loneliness is a negative or distressing emotion that accompanies the perception that one's social needs are not being fully met and may follow different developmental trajectories (36). It can become a negative factor of satisfaction with life and mental wellbeing if the experience of loneliness becomes chronic (37). Our research results also show that adolescents' self-esteem is associated with life satisfaction and mental wellbeing. A similar trend was reported in previous studies (38, 39). One paper indicated that high self-esteem can predict mental health conditions, where adolescents with higher self-esteem are linked to fewer psychiatric symptoms such as anxiety/depression (38, 39). It is thus necessary to educate families to be aware of the importance of self-esteem and adapt interventions to increase the self-esteem of their children. In terms of relationships within the family, conflicts with the father have been found to negatively affect the life satisfaction and mental wellbeing of adolescents. This finding is in line with a study by Lachowska (40), which suggests that the compromise and conflict resolution of a father and his adolescent child may have a positive effect on the life satisfaction of that child. Negative impacts of troublesome relationships with parents can also severely inhibit the mental health development of adolescents (41). Similarly, having support and close relationships with family members was positively associated with the youth's life satisfaction and mental wellbeing. Strengthening the emotional bond of the family as well as encouraging teenagers to talk about their problems, therefore, are important implications to improve the life satisfaction and mental wellbeing of adolescents (6, 42).

In terms of protective factors, individuals, families, and peers positively predict the resilience of participants, which increased the life satisfaction of adolescents. Previous studies have shown the correlation of different variables, such as individual characteristics (gender, grade), self-esteem, resilience, conflict with parents, loneliness, and isolation within their family, having supportive and sharing family members, with life satisfaction and mental wellbeing (14, 16, 19, 20, 33, 40, 42–44). However, our study proposes a new model of evaluating factors associated with adolescents' life satisfaction and mental wellbeing. This predictive model indicates that individual factors (including grade and gender) and conflict with parents negatively affect adolescents' life satisfaction and mental wellbeing. Besides, self-esteem and protective factors for resilience are two important mediating factors between feelings of students at home (including loneliness and isolation within the family and having supportive and sharing family members), conflict with parents, and life satisfaction and mental wellbeing.

Our research once again highlights the impact of family on life satisfaction and the mental wellbeing of children. Not only at school, but at home, children should be encouraged to share their feelings and resolve conflicts with their parents. Our research lays the groundwork for multi-level analysis for simultaneous assessments of personal and interpersonal factors to address remaining problems and promote children's wellbeing and life satisfaction. From this ecological framework, interventions must focus on strengthening social support across all dimensions of the children's lives, community resources, and family relationships.

Our study has certain limitations. Firstly, the cross-sectional study model prevented us from determining a causal relationship. Secondly, the information was collected from self-reported questionnaires, which may be subject to personal bias, such as academic performance may be subjective. Thirdly, although the Satisfaction with Life Scale (SWLS) is used worldwide, no previous studies have been conducted in Vietnam and therefore we could not provide a comparison of findings within the country. However, comparisons with global studies were conducted. Despite the above limitations, our research is among the pioneers in providing a comprehensive view of life satisfaction and mental wellbeing in adolescents by exploring individual and community-related factors, including social relationships.

## Conclusion

Our study investigates important factors associated with life satisfaction and mental wellbeing among adolescents, specifically parent-child relationships, self-esteem, and resilience. The results highlight the influences of academic expectations and gender bias on the mental wellbeing of youths, especially in Asian cultures. Strengthening the family bond and encouraging young people to share their feelings are crucial to enhancing adolescents' mental health status.

## Data availability statement

The original contributions presented in the study are included in the article/supplementary material, further inquiries can be directed to the corresponding author.

## Ethics statement

The studies involving human participants were reviewed and approved by all procedures performed in studies involving human participants were by the ethical standards of the Youth Research Institute, Vietnam, and with the 1964 Helsinki Declaration and its later amendments or comparable ethical standards. Online Informed consent was obtained from all participants. Written informed consent to participate in this study was provided by the participants' legal guardian/next of kin.

## Author contributions

Survey conception: VT, HND, and TMT. Data collection: VT, TBT, KV, HMD, and NT. Project management: VT and TPT. Data analysis: TN and VT. Data interpretation: HND and TMT. Drafting of the manuscript: VT. Reviewing and editing the manuscript: PA, LB, GF, CL, CH, and RH. All authors revised the manuscript critically and gave final approval of the version to be published.
